# Low-Intensity Extracorporeal Shock Wave Therapy Ameliorates the Overactive Bladder: A Prospective Pilot Study

**DOI:** 10.1155/2020/9175676

**Published:** 2020-07-06

**Authors:** Yung-Chin Lee, Shu-Mien Chuang, Kun-Ling Lin, Wei-Chiao Chen, Jian-He Lu, Kuang-Shun Chueh, Mei-Chen Shen, Li-Wen Liu, Cheng-Yu Long, Yung-Shun Juan

**Affiliations:** ^1^Department of Urology, College of Medicine, Kaohsiung Medical University, Kaohsiung, Taiwan; ^2^Department of Urology, Kaohsiung Municipal Hsiao-Kang Hospital, Kaohsiung, Taiwan; ^3^Department of Urology, Kaohsiung Medical University Hospital, Kaohsiung, Taiwan; ^4^Translational Research Center, Cancer Center, Department of Medical Research, Kaohsiung Medical University, Kaohsiung, Taiwan; ^5^Department of Obstetrics and Gynecology, Kaohsiung Medical University Hospital, Kaohsiung, Taiwan; ^6^Graduate Institute of Clinical Medicine, College of Medicine, Kaohsiung Medical University, Kaohsiung, Taiwan; ^7^Department of Obstetrics and Gynecology, Kaohsiung Municipal Ta-Tung Hospital, Kaohsiung, Taiwan; ^8^Department of Urology, Kaohsiung Municipal Ta-Tung Hospital, Kaohsiung, Taiwan; ^9^Department of Obstetrics and Gynecology, Kaohsiung Municipal Hsiao-Kang Hospital, Kaohsiung, Taiwan; ^10^Graduate Institute of Medicine, College of Medicine, Kaohsiung Medical University, Kaohsiung, Taiwan

## Abstract

**Objective:**

In the present clinical trial, we evaluated the therapeutic effects of low-intensity extracorporeal shockwave therapy (LiESWT) on overactive bladder (OAB).

**Methods:**

Female subjects with ages of 20-75 years and who have been clinically diagnosed with OAB were included in the study. The LiESWT (DUOLITH SD1 T-TOP, AG) applicator was placed on the suprapubic skin area and applied with an intensity of 0.25 mJ/mm^2^, 3000 pulses, and 3 pulses/second. To assess the therapeutic efficacy, all subjects were required to complete the validated OAB symptoms and life bothersome questionnaires, 3-day urinary diary, uroflowmetry, and post-voided residual urine (PVR) measurement at 4 weeks of LiESWT (W4), 8 weeks of LiESWT (W8), 1-month follow-up (F1), and 3-month follow-up (F3) after LiESWT.

**Result:**

82 subjects with the mean age of 56.5 ± 1.2 years were enrolled. The questionnaire scores were significantly improved at W4, W8, F1, and F3 as compared to baseline data (W0). At W8, the mean values of functional bladder capacity were meaningfully increased. According to the 3-day urinary diary, daytime frequency, urgency, and nocturia were significantly decreased. The uroflowmetry results showed that the mean voided urine volume and the maximal flow rate (*Q*_max_) were noticeably increased. PVR volume was also significantly decreased.

**Conclusions:**

The data demonstrated that 8-week LiESWT ameliorated the OAB symptoms, promoted the uroflow parameters, and improved the quality of life (QoL) in OAB patients, suggesting that LiESWT might serve as an alternative noninvasive therapy for OAB.

## 1. Introduction

An overactive bladder (OAB) is a prevalent condition, which can seriously reduce overall quality of life (QoL). It is a major financial burden on healthcare with prevalence rates of 16-17%. In Asian countries, human epidemiology studies revealed that the prevalence of female OAB is 20.9% and is age-dependent; it increases to 34.5% in those who are over the age of 65 [1–3]. The physiological basis of these symptoms is detrusor overactivity. Previous studies revealed that hypoxia, oxidative stress, and a decrease of blood supply play a role in pathophysiology of OAB symptoms [4, 5]. Changes in bladder innervation, a disproportionate increase of prostaglandin by muscarinic as well as purinergic receptors, and leukotriene production may also be involved in the development of OAB [6, 7].

Currently, there are some treatment options available for OAB patients. The clinically used medical treatment for OAB includes antimuscarinic agents and/or*β*3 agonist medication [8, 9]. However, the use of medication, especially anticholinergic agents, is frequently accompanied with adverse effects, such as dry mouth, constipation, and blurred vision. Side-effects of *β*3 agonist medication agents include changes in blood pressure, dizziness, and somnolence [8, 9]. Whenever medication treatment fails, invasive treatment with intravesical botulinum toxin A injection and posterior tibial nerve stimulation (PTNS) or sacral neuromodulation (SNM) are considered as third-line treatment [10, 11]. The effect of botulinum toxin A is to block the presynaptic release of acetylcholine from the parasympathetic efferent nerve. However, the efficacy might have some effects on altering the afferent nerve input, which results in acute urinary retention, large post-void residual (PVR) (>150 ml), and gross hematuria [12]. Although PTNS and SNM present a safer and short-term improvement, both are invasive procedures which need accurate needle puncture into the proper position [13]. Moreover, it takes 3 months to determine the success of PTNS [13]. As a result of these findings, the current therapies for OAB are still limited in that they are designed to mitigate symptoms rather than treat the underlying pathology. Moreover, the side-effects of the therapies are the major cause for the low persistent rate of medical treatment. Therefore, finding an alternative treatment, especially a nonmedical and less invasive therapy without disturbing adverse effects, is of utmost important for OAB patients.

Low-intensity extracorporeal shockwave therapy (LiESWT) has been used as a clinical treatment modality in many types of diseases, including myocardial infarction, heart failure [14], bone fracture, tendinopathy [15], and skin ischaemia [16]. LiESWT has also been proven to be an effective and safe treatment for various urological disorders, including chronic pelvic pain syndrome (CPPS) [17–21] and erectile dysfunction (ED) [22–26]. Clinically, LiESWT (0.10-0.25 mJ/mm^2^, 3000 pulses, once/week, 4 weeks) improves pain score, bladder voiding, and QoL and could be considered as a treatment strategy for CPPS. Moreover, the therapeutic effects of LiESWT (0.10-0.25 mJ/mm^2^, 3000-6000 pulses, once/week, 4-8 weeks) for ED patients increased penile hemodynamics and induced penile tissue regeneration. A systemic review revealed that LiESWT can improve erectile function, as measured by the International Index of Erectile Function (IIEF) and Erectile Hardness Score (EHS) [27].

Recently, our group showed that LiESWT can significantly improve the visual analog scale (VAS) pain score and total international prostate symptom scores (IPSS) for men with CPPS refractory to 3-As therapy (antibiotics, alpha-blockers, and anti-inflammatories). The response can last until the 3-month follow-up [19]. To date, the clinical efficacy of LiESWT on patients with OAB has not previously been investigated. Our research was the first clinical study to investigate the therapeutic effect of LiESWT on OAB. We hypothesized that LiESWT could ameliorate detrusor overactivity by attenuating the inflammatory responses of the bladder, which consequently could alleviate OAB symptoms. The aim of this study was to evaluate the effect of LiESWT on clinical symptoms, urodynamic parameter, and QoL of OAB subjects.

## 2. Materials and Methods

### 2.1. Eligibility of Subject

This clinical trial was a prospective pilot study, which was performed with the approval of the institutional review board of a tertiary medical center (Kaohsiung Medical University Hospital, Kaohsiung, Taiwan; clinical trial registration No. KMUHIRB-F(II)-20180010). This study was also registered at ClinicalTrials.gov (NCT04059133), and the date of registration was August 16, 2019. Eligible participants were recruited from the outpatient clinics and provided informative consent before participating in the study. All participants had a complete medical history and physical examination. This study included female subjects (participants) aged 20-75 years who were diagnosed with OAB for more than 3 months, such as daytime frequency of micturition ≥ 8 times, nocturia ≥ 1 times, urgency ≥ 1 times, or urgency incontinence ≥ 1 times. Major exclusion criteria are as follows: (1) urinary tract infection (UTI) at screening period or recurrent UTI (defined as ≥3 UTIs in the previous 3 months); (2) neuropathic diseases; (3) history of urolithiasis or urologic cancer; (4) lower urinary tract surgery in the last 6 months; (5) severe cardiopulmonary disease, coagulopathy, and liver or renal dysfunction; (6) gross hematuria; (7) significant bladder outflow obstruction; (8) previous pelvic radiation therapy; and (9) drug or nondrug treatments of OAB in the previous 3 months. No other urinary-related treatments were permitted during the study and follow-up periods. Women who are pregnant or within six months of childbearing are also excluded from the study.

### 2.2. Medical Information of LiESWT

**Figure 1:**
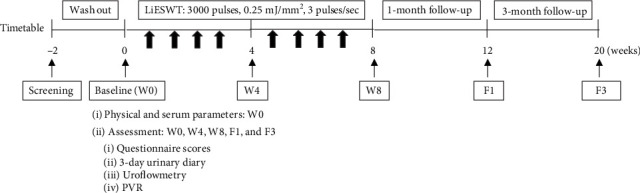
Flow chart for the LiESWT treatment procedure. LiESWT: low-intensity extracorporeal shock wave therapy; PVR: measurement of post-voided residual urine volume; OABSS: overactive bladder symptom scores; ICIQ-SF: international consultation on incontinence questionnaire-short form; UDI-6: urogenital distress inventory-short form; IIQ-7: incontinence impact questionnaire-7.

The instrumentation was the DUOLITH SD1 T-TOP focused shock wave system (Storz Medical AG). The LiESWT was applied with an intensity 0.25 mJ/mm^2^, the number of shocks = 3000 pulses, and a frequency of 3 pulses/second as modified from previous reports [28]. Participants were required to rest on a treatment couch for 15 minutes before treatment. Experienced urologists and gynecologists gently placed the applicator on the suprapubic skin area over the bladder dome (1000 impulses) and over the bilateral bladder walls (each side 1000 impulses). The probe was placed on the lower abdomen with two fingers apart from the pubic symphysis, tilting to 45° (Figure 1).

### 2.3. Physical and Serum Biochemical Parameters of Study Subjects

The physical and serum parameters of metabolic syndrome were associated with the symptoms of OAB. Physical indicators were collected, including height, weight, waistline, body mass index (BMI), and blood pressure. Additionally, serum biochemical parameters were examined, including hemoglobin A1c (glycated hemoglobin; HbA1c), blood sugar before meals, renal function index (blood urea nitrogen (BUN)), liver function index (glutamate oxaloacetate transaminase (GOT), glutamate pyruvate transaminase (GPT)), and lipid profile (triglycerides, cholesterol, low-density lipoprotein (LDL), and high-density lipoprotein (HDL)) to investigate the baseline characteristics of the OAB population.

### 2.4. Uroflowmetry and Measurement of PVR Urine Volume

Uroflowmetry served as a noninvasive screening test. Before LiESWT, uroflowmetry and PVR amount were checked to rule out voiding dysfunction. For uroflowmetry, voided urine volume and maximum flow rate (*Q*_max_) were recorded. The amount of PVR was calculated by the Verathon BladderScan BVI 9400 (Radiance Medical Systems, Kuala Lumpur, Malaysia). Uroflowmetry and PVR helped to identify subjects in need of further evaluation and to evaluate treatment effect during follow-up.

### 2.5. Therapeutic Efficacy Assessment for LiESWT

The researchers received training on instrument operation, diagnostic confirmation, disease activity assessment, data key in, and data quality control. To analyze the therapeutic efficacy assessment for LiESWT, the primary endpoint was changed in the questionnaire scores, and the secondary endpoints were changed in the 3-day urinary diary and uroflowmetry at baseline (W0), 4 weeks (W4) of LiESWT, 8 weeks (W8) of LiESWT, 1-month follow-up (F1), and 3-month follow-up (F3) after LiESWT. The completed 3-day urinary diary chart will provide subjects' information about average fluid intake, urine output, functional bladder capacity, daytime frequency, urgency, and nocturia over a period of three days. Moreover, validated questionnaires included the overactive bladder questionnaire short form (Overactive Bladder Symptom Scores (OABSS)) [29], International Consultation on Incontinence Questionnaire-Short Form (ICIQ-SF) [30], Urogenital Distress Inventory-6 (UDI-6-) Short Form, and Incontinence Impact Questionnaire-7 (IIQ-7) score [31]. Uroflowmetry and PVR measurement were also performed to assess the urodynamic parameters at W0, W4, W8, F1, and F3. The primary endpoint was the mean change in OAB symptoms and urodynamic parameters from baseline to the end of treatment.

### 2.6. Statistical Analysis

Questionnaires, uroflowmetry, and 3-day urinary diary were used to assess the efficacy of the pre- and posttreatments performed with LiESWT on OAB subjects. Quantitative data were represented as mean ± standard error (SE). In order to clarify the effect of LiESWT therapy on OAB, we compared the pre- and posttreatment scores (W4 vs. W0, W8 vs. W0, F1 vs. W0, and F3 vs. W0) for an intragroup of patients, not between groups of patients. Therefore, a paired *t*-test was used to perform a repeated measurement analysis for an intragroup before/after treatment and to calculate *p* values for comparison [32, 33] in a single-arm clinical trial of the current study. For all statistical analyses, *p* < 0.05 was considered to be statistically significant. All statistical analyses were performed using SAS 9.3 (SAS Institute, Cary, NC, USA).

## 3. Results

### 3.1. Characteristics of Study Subjects

**Figure 2:**
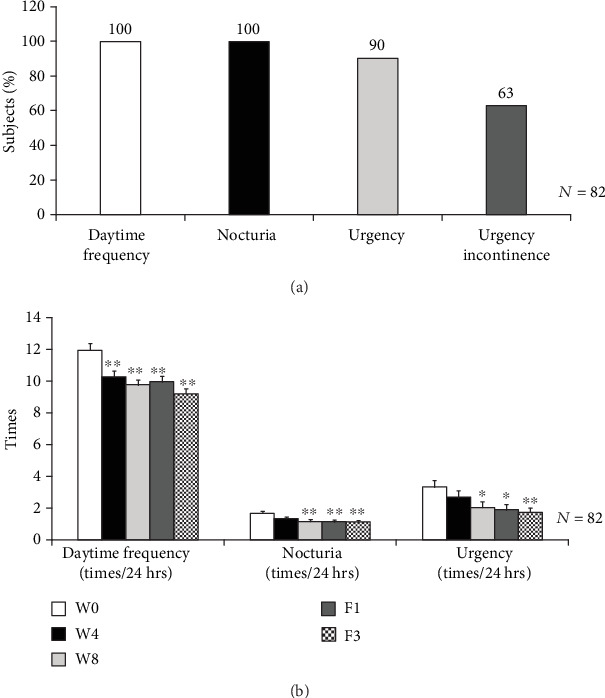
Analysis of the study population with OAB symptoms. (a) The percentage of the study population with OAB symptoms. OAB consisted of urinary urgency with or without urgency incontinence, often accompanied by daytime frequency and nocturia. (b) The changes in daytime frequency, nocturia, and urgency at W4, W8, F1, and F3. ^∗^*p* < 0.05; ^∗∗^*p* < 0.01 compared to baseline (W0). W4: 4 weeks of LiESWT treatment; W8: 8 weeks of LiESWT treatment; F1: 1-month follow-up; F3: 3-month follow-up.

The physical indicators and biochemical parameters of the OAB subjects were summarized in Table 1. The mean age of the enrolled 82 subjects was 56.5 ± 1.2 years. The percentages of subjects with daytime frequency and nocturia were 100%. The percentages of subjects with urgency and urgency incontinence were 90% and 63% in Figure 2(a). All subjects completed treatment and follow-up periods.

### 3.2. LiESWT Improved 3-Day Urinary Diary

The analysis of the 3-day urinary diary was characterized in Figure 2(b) and Table 2. There was no significant difference in the amount of fluid intake and urine output among different visits. However, the mean values of functional bladder capacity were noticeably increased from 325.28 ± 15.04 ml to 366.94 ± 12.83 ml at W8. LiESWT also showed a significant increase in the average voided volume at W8, F1, and F3. With regards to OAB symptoms, the daytime frequency values were meaningfully decreased from 11.94 ± 0.45 (W0) to 10.25 ± 0.38 (W4) and 9.76 ± 0.34 (W8). Nocturia was significantly decreased from 1.67 ± 0.15 (W0) to 1.16 ± 0.12 (W8). The mean times of the urgency were decreased from 3.33 ± 0.41 (W0) to 2.04 ± 0.35 (W8). Moreover, all the mean values of daytime frequency, urgency, and nocturia remained significantly decreased at F1 and F3.

### 3.3. LiESWT Improved Uroflowmetry Parameters and PVR

The analysis of urodynamic parameters was determined using uroflowmetry (voided urine volume and *Q*_max_) and PVR as shown in Table 2. In the uroflowmetry study, the mean of voided urine volume (ml) were meaningfully increased from 301.43 ± 15.23 (W0) to 349.69 ± 17.45 (W4) and 364.42 ± 17.99 (W8). The mean of *Q*_max_ (ml/sec) was significantly increased from 24.92 ± 1.18 (W0) to 28.43 ± 1.13 (W8). The PVR (ml) also had a significant decrease from 49.8 ± 5.5 (W0) to 33.8 ± 4.2 (W8). Moreover, the mean of voided urine volume and PVR remained noticeably improved at F1 and F3. These results indicated that eight weeks of LiESWT caused significant improvements in voided urine volume, *Q*_max_, and PVR.

### 3.4. LiESWT Improved OAB Symptoms and QoL

**Figure 3:**
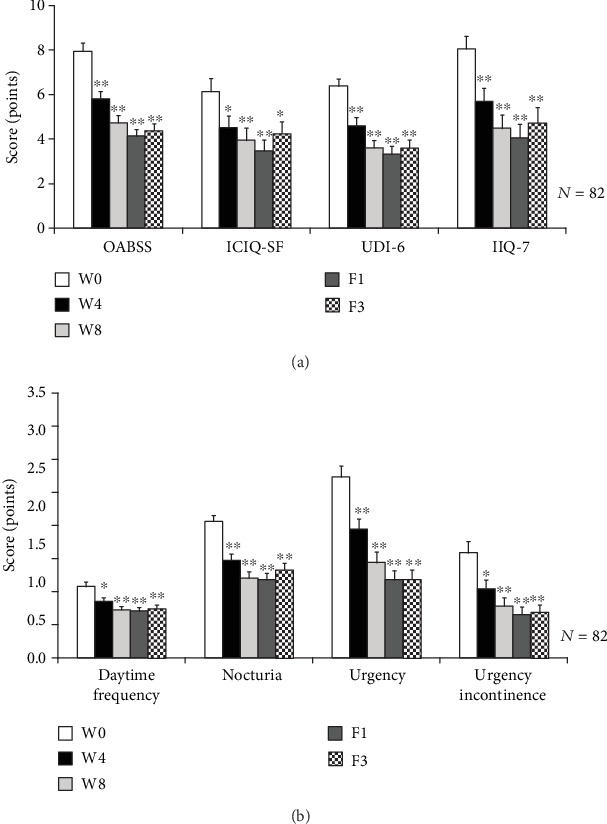
The change of OAB symptoms and bothersome questionnaire scores after LiESWT treatment. (a) The bothersome questionnaire scores included overactive bladder (OAB) symptom scores (OABSS), international consultation on incontinence questionnaire-short form (ICIQ-SF), urogenital distress inventory-6- (UDI-6-) short form, and incontinence impact questionnaire-7 (IIQ-7) score at W4, W8, F1, and F3. (b) The improvement of questionnaire scores for overactive bladder (OAB) symptom after LiESWT treatment, including daytime frequency, nocturia, urgency, and urgency incontinence. ^∗^*p* < 0.05; ^∗∗^*p* < 0.01 compared to baseline (W0). W4: 4 weeks of LiESWT treatment; W8: 8 weeks of LiESWT treatment; F1: 1-month follow-up; F3: 3-month follow-up.

After 8 weeks of LiESWT, the OAB symptoms and bothersome questionnaire scores (OABSS, ICIQ-SF, UDI-6, and IIQ-7) were investigated (Table 2 and Figure 3). The OABSS score was noticeably decreased from 7.9 ± 0.4 (W0) to 5.8 ± 0.3 (W4) and 4.7 ± 0.3 (W8). The ICIQ-SF score was significantly decreased from 6.2 ± 0.6 (W0) to 4.5 ± 0.5 (W4) and 3.9 ± 0.6 (W8). The UDI-6 score was decreased from the baseline 6.4 ± 0.3 (W0) to 4.6 ± 0.4 (W4) and 3.6 ± 0.3 (W8). The IIQ-7 score was decreased from the baseline 8.1 ± 0.6 (W0) to 5.7 ± 0.6 (W4) and 4.5 ± 0.6 (W8). All the questionnaire scores (OABSS, ICIQ-SF, UDI-6, and IIQ-7) remained significantly decreased at F1 and F3. Our results indicated that eight weeks of LiESWT caused significant improvements in OAB symptoms and QoL.

According to the subgroups of OABSS (Table 2 and Figure 3(b)), daytime frequency was significantly decreased from 1.1 ± 0.1 (W0) to 0.9 ± 0.1 (W4) and 0.8 ± 0.1 (W8). Nocturia was meaningfully decreased from 2.2 ± 0.1 (W0) to 1.5 ± 0.1 (W4) and 1.3 ± 0.1 (W8). The mean time of the urgency was decreased from the baseline 2.9 ± 0.2 (W0) to 2.0 ± 0.2 (W4) and 1.5 ± 0.2 (W8). The mean time of the urgency incontinence was decreased from the baseline 1.7 ± 0.2 (W0) to 1.1 ± 0.1 (W4) and 0.8 ± 0.1 (W8). All the subgroups of OAB symptom scores remained significantly decreased at F1 and F3.

### 3.5. Safety of LiESWT Treatment

In this study, treatments were well tolerated by the subjects. No significant adverse effects associated with LiESWT, such as intolerable pain, hematuria, or skin ecchymosis, were reported from subjects.

### 3.6. Short Graphic Abstract of Study Proposed for Potential Effect of LiESWT

**Figure 4:**
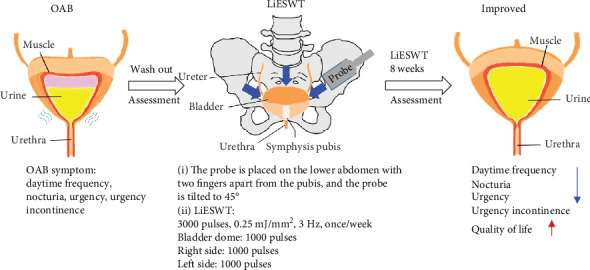
Short graphic abstract of the study as proposed for the potential effect of LiESWT. OAB: overactive bladder; LiESWT: low-intensity extracorporeal shock wave therapy.

The above findings led to the suggestion of a model to postulate the potential effect of LiESWT on OAB, as shown in Figure 4. The LiESWT was applied with a 0.25 mJ/mm^2^ intensity, 3000 pulses of shocks, and a frequency of 3 pulses/second, and was placed on the lower abdomen with two fingers apart from the pubis, tilting the probe to 45°. The results revealed that LiESWT reduced the symptoms of OAB, including urinary incontinence, urgency, frequency, and nocturia after 8 weeks of treatment, which brought significant improvement in QoL.

## 4. Discussion

LiESWT has been clinically used in the treatment of many diseases, but the therapeutic effect of LiESWT on OAB had never been explored. The present investigation was the first clinical study to demonstrate that 8 weeks of LiESWT treatment could ameliorate OAB symptoms; improve the urodynamic parameters, such as voided urine volume, *Q*_max_, and PVR; and increase functional bladder capacity, which brought significant improvement in QoL (Figure 4).

LiESWT has been used clinically for several years in urology, but the efficacy and safety profile of LiESWT in treating OAB are not completely clear. For the CPPS studies, the shock intensity ranging from 0.10 to 0.25 mJ/mm^2^, 3000 impulses, and once/week treatment continuing for 4 weeks were used to treat the disease [17–19]. The results showed that LiESWT improved the pain score, bladder urination, and QoL without side-effects. On the other hand, ED studies were treated with 0.10-0.25 mJ/mm^2^, 3000-6000 pulses, and once per week treatment continuing for 4-8 weeks [22–24, 34]. Evidences suggested that LiESWT treatment induced penile tissue regeneration and increased penile hemodynamics. Gruenwald et al. determined the efficacy of LiESWT in severe ED patients who were poor responders to PDE5i therapy, and their results suggested that LiESWT significantly improved the symptoms [34]. In our study, the recruited subjects had normal physical and serum parameters, thus eliminating potential confounding factors (Table 1). Our findings demonstrated that OAB symptoms and QoL had persistent improvement at W8, F1, and F3 after LiESWT treatment (0.25 mJ/mm^2^, 3000 pulses, 3 pulses/second, and once/week); however, the effectiveness of different treatment intervals and frequencies must be further evaluated in order to determine the optimal treatment protocol of LiESWT for OAB patients.

The molecular mechanism underlying the therapeutic effect of LiESWT is still unclear, but it is difficult to obtain bladder tissues in clinical trials. Hence, investigating the molecular mechanism of the therapeutic effect of LiESWT through animal experiments is necessary. Zhang et al. reported that the shock intensity ranging from 0.10 to 0.13 mJ/mm^2^ and the shock number ranging from 200 to 300 impulses were the optimal parameters for LiESWT to increase angiogenetic factors and decrease inflammation mediators [35]. In rats with cyclophosphamide-induced acute interstitial cystitis [36], LiESWT (200 impulses at 0.11 mJ/mm^2^) was found to improve bladder overactivity through inducing angiogenesis, inhibit the production of reactive oxygen species (ROS), and lessen oxidative stress. Wang et al. reported that LiESWT was applied for 4 weeks once a week using a 0.02 mJ/mm^2^ energy flux density at 3 Hz for 400 pulses which ameliorated diabetic bladder dysfunction and urinary incontinence in a streptozotocin-induced diabetic rat. These studies indicated that LiESWT ameliorated bladder wall composition, enhanced bladder and urethra muscle contractile function, increased bladder nerve innervation, activated bladder muscle regeneration, and promoted urethra continence [37]. Another study was on diabetic bladder dysfunction also induced by streptozotocin, which showed that LiESWT (300 impulses at 0.1 mJ/mm^2^, 3 times/week, 4 weeks) enhanced nerve innervation and vascularization of the bladder, and improved bladder function, such as daytime frequency, urine volume per void, and PVR in endogenous stem cells [38].

It is well known that the lower urinary tract symptom is related to female sexual function. Females with an overactive bladder and urinary incontinence had a worsened sexual life, decreased sexual desire, and loss of arousal [39, 40]. From previous literature, estrogen supply can not only improve the symptoms of an overactive bladder and urinary incontinence but also increase sexual life [40–42]. In our study, LiESWT had an obvious benefit on lower urinary tract symptoms. Therefore, LiESWT might play an important role in treating female sexual dysfunction, especially in combination with estrogen therapy in postmenopausal women. This is a new topic worthy of further research.

Due to the lack of molecular investigations, the present study could not provide evidence about the potential therapeutic mechanisms of the effect of LiESWT on OAB. However, our results provided new insights into potential therapies, which might lead to a new alternative treatment for OAB patients. It should be pointed out that the current study had several limitations. First, it was a prospective pilot study, which lacked control or comparison with other types of therapy. Second, the patient number was limited; hence, a comparison of the efficacy of LiESWT with different symptoms of OAB was not feasible. Third, the follow-up period was short, only one month and 3 months; hence, the long-term and persistence of efficacy after LiESWT treatment could not be evaluated. Fourth, only female participants were included in this study, so the therapeutic efficacy of LiESWT on male subjects may require further research.

## 5. Conclusion

The present study showed that 8 weeks of LiESWT could ameliorate the OAB symptoms, improve QoL, and enhance urodynamic parameters of OAB patients. LiESWT might serve as a new potential noninvasive alternative therapy in patients with OAB. Further larger and longer-term investigations are needed to validate the results in the present study and to optimize the therapeutic efficacy of LiESWT on OAB.

## Figures and Tables

**Table 1:** Baseline characteristics of overactive bladder (OAB) population.

Parameter	OAB (mean ± SE)	Range
Physical parameter		
Female age (years)	56.5 ± 1.2	20-75
Height (cm)	157.52 ± 0.53	
Weight (kg)	57.89 ± 0.88	
BMI (kg/m^2^)	23.33 ± 0.34	18.5-24
Waistline (cm)	83.77 ± 1.03	
Systolic pressure (mmHg)	122.9 ± 2.5	100-120
Diastolic pressure (mmHg)	73.0 ± 1.5	60-80
MAP (mmHg)	89.62 ± 1.73	70-110
Serum parameter		
HbA1c (%)	5.66 ± 0.10	4-6
AC sugar (mg/dl)	102.1 ± 2.7	65-109
BUN (mg/dl)	12.22 ± 0.47	8-20
Creatinine (mg/dl)	0.71 ± 0.02	0.44-1.03
GOT (AST) (IU/l)	25.3 ± 1.4	10-42
GPT (ALT) (IU/l)	23.2 ± 1.4	10-40
Triglycerides (mg/dl)	109.6 ± 8.9	35-160
Cholesterol (mg/dl)	206.3 ± 4.8	140-200
HDL (mg/dl)	59.69 ± 1.92	29-85
LDL (mg/dl)	122.66 ± 3.84	0-130

Note: BMI: body mass index; MAP: mean arterial pressure; HbA1c: hemoglobin A1c (glycated hemoglobin); AC: ante cibum (before meals); BUN: blood urea nitrogen; GOT: glutamate oxaloacetate transaminase; GPT: glutamate pyruvate transaminase; HDL: high-density lipoprotein; LDL: low-density lipoprotein. Values are means ± SE. *N* = 82.

**Table 2:** Urodynamic parameters of study population for overactive bladder (OAB).

Parameter	OAB (mean ± SE)				
	W0	W4	W8	F1	F3
3-Day urinary diary record					
Intake (ml)	1868.06 ± 81.94	1792.07 ± 76.35	1977.92 ± 180.65	1761.80 ± 82.47	1766.56 ± 74.50
Output (ml)	2045.26 ± 87.63	1922.54 ± 75.81	1909.54 ± 75.78	1928.33 ± 74.51	1819.03 ± 77.33
Average voided volume (ml)	182.51 ± 8.20	195.02 ± 8.03	206.93 ± 8.94^∗^	215.67 ± 12.10^∗^	208.77 ± 8.88^∗^
Functional bladder capacity (ml)	325.28 ± 15.04	338.03 ± 12.01	366.94 ± 12.83^∗^	359.49 ± 10.87	359.55 ± 13.72
Daytime frequency (times)	11.94 ± 0.45	10.25 ± 0.38^∗∗^	9.76 ± 0.34^∗∗^	9.70 ± 0.30^∗∗^	9.19 ± 0.34^∗∗^
Nocturia (times)	1.67 ± 0.15	1.34 ± 0.12	1.16 ± 0.12^∗∗^	1.14 ± 0.11^∗∗^	1.11 ± 0.11^∗∗^
Urgency (times)	3.33 ± 0.41	2.68 ± 0.43	2.04 ± 0.35^∗^	1.89 ± 0.34^∗^	1.72 ± 0.30^∗∗^

Uroflowmetry data					
Voided urine volume (ml)	301.43 ± 15.23	349.69 ± 17.45^∗^	364.42 ± 17.99^∗^	350.69 ± 15.91^∗^	355.80 ± 16.15^∗^
Maximum flow rate (*Q*_max_ ) (ml/sec)	24.92 ± 1.18	26.05 ± 1.39	28.43 ± 1.13^∗^	29.08 ± 1.22^∗^	31.25 ± 2.65^∗^
Post-voided residual (PVR) (ml)	49.8 ± 5.5	41.2 ± 4.9	33.8 ± 4.2^∗^	28.9 ± 3.3^∗∗^	32.0 ± 2.9^∗^

OABSS score (points)					
Daytime frequency	1.1 ± 0.1	0.9 ± 0.1^∗^	0.8 ± 0.1^∗∗^	0.7 ± 0.1^∗∗^	0.8 ± 0.1^∗∗^
Nocturia	2.2 ± 0.1	1.5 ± 0.1^∗∗^	1.3 ± 0.1^∗∗^	1.2 ± 0.1^∗∗^	1.4 ± 0.1^∗∗^
Urgency	2.9 ± 0.2	2.0 ± 0.2^∗∗^	1.5 ± 0.2^∗∗^	1.2 ± 0.2^∗∗^	1.2 ± 0.1^∗∗^
Urgency incontinence	1.7 ± 0.2	1.1 ± 0.1^∗^	0.8 ± 0.1^∗∗^	0.7 ± 0.1^∗∗^	0.7 ± 0.1^∗∗^

*Note*. SE: standard error; W: week; W4: once per week, 4 weeks of LiESWT; W8: once per week, 8 weeks of LiESWT; F1: 1-month follow-up; F3: 3-month follow-up; OABSS: overactive bladder symptom scores. Values are means ± SE. ^∗^*p* < 0.05; ^∗∗^*p* < 0.01 vs. W0. *N* = 82.

## Data Availability

The data used to support the findings of this study are included within the article.
